# Grandparent involvement and children’s emotional and behavioral problems: the mediating role of social competence and the moderating role of family functioning

**DOI:** 10.3389/fpsyg.2026.1851087

**Published:** 2026-09-25

**Authors:** Yanzhen Xu, Xiuting Wen, Yumeng Hao, Huiyan Lin, Yakun Ni

**Affiliations:** 1 School of Educational Science, Guangdong Polytechnic Normal University, Guangzhou, China; 2 Department of Public Administration, Guangdong University of Finance, Guangzhou, China

**Keywords:** emotional and behavioral problems, family functioning, grandparent involvement, moderated mediation, social competence

## Abstract

This study examined whether social competence accounts for part of the association between grandparent involvement and children’s emotional and behavioral problems, and whether family functioning modifies the strength of the association between grandparent involvement and social adaptability. Data were collected from 729 parents of children aged 3–7 years in China across three time points. Participants completed measures of grandparent involvement, family functioning, social adaptability, and emotional and behavioral problems. Moderated mediation analysis revealed that grandparent involvement was positively associated with children’s emotional and behavioral problems, and social adaptability partially accounted for this association. Moreover, the negative association between grandparent involvement and social adaptability varied by family functioning: the negative association was weaker when family functioning was high and stronger when family functioning was low. These findings suggest that family functioning and children’s social adaptability may serve as promising intervention targets for addressing the negative correlates of grandparent involvement with child development.

## 1 Introduction

Against the backdrop of an aging population, grandparent involvement has emerged as a pervasive supplementary form of childcare in Chinese families, wherein grandparents assume partial or full responsibility for raising their grandchildren (Fuller-Thomson and Minkler, 2001; Dommaraju and Wong, 2025). Unlike Western “custodial grandparenting” which frequently arises from parental crises (Kaylor-Tapscott and Sullivan, 2024), grandparent involvement in contemporary China represents a normative, voluntary, and collaborative “intergenerational care collaboration” arrangement rooted in the Confucian tradition of Filial Piety (Xiao) (Chen et al., 2025).

While extensive grandparent involvement offers substantial instrumental and logistical relief to working parents (Buchanan and Rotkirch, 2018), high levels of grandparent involvement are associated with greater structural complexity to daily child-rearing environments (Qiu and Shum, 2022). In particular, when grandparents assume a high frequency and long duration of daily care, the sheer quantity of adult-child interactive episodes within the household intensifies substantially. Theoretically, higher grandparent involvement intensity may correspond to reduced temporal availability of parents for exclusive one-on-one emotional communication, shared activities, and sensitive responsiveness. Previous research suggests that such a reduction could be associated with diluted functional salience of parents as primary attachment figures and with compromised children’s acquisition of emotion regulation capacities (Groh et al., 2017; Dagan and Sagi-Schwartz, 2018) and with compromised children’s acquisition of emotion regulation capacities (Prime et al., 2020). In line with this, recent evidence suggests that declines in unstructured, self-directed activities are associated with poorer self-regulation (Colliver et al., 2022; Gray et al., 2023). Moreover, children in high-intensity grandparent involvement contexts spend proportionally more time within adult-monitored domestic settings, and the cumulative frequency of unstructured, self-directed micro-moments (during which children naturally practice frustration tolerance, delay of gratification, and independent emotional recovery) is objectively lower (Barker et al., 2014). Reduced parental emotional availability and diminished autonomous coping behaviors are associated with children’s behavioral problems (Luo et al., 2020). Crucially, the extent to which high caregiving involvement relates to child adjustment varies systematically across household contexts, highlighting overall family functioning as a potential moderator of this association (Mendoza and Lee, 2023).

Existing research, however, still manifests distinct limitations. First, there is a lack of in-depth exploration into the specific underlying indirect link mechanisms through which grandparent involvement co-varies with children’s emotional and behavioral problems. Second, most studies remain at the level of phenomenological description, with few investigating how to mitigate the potential negative consequences of grandparent involvement from the perspective of family functioning.

Beyond direct associations, individual-level psychological pathways connecting household grandparent involvement structures to child behavioral outcomes warrant further articulated examination. During early childhood, children’s social competence, defined as the integrated capacity to initiate and maintain positive peer relationships, resolve interpersonal conflicts constructively, regulate emotions in social contexts, and apply prosocial strategies in extra-familial settings, represents a core developmental competency that is associated with long-term psychosocial adjustment (Rubin et al., 2006; Denham et al., 2003; Zhang et al., 2022). From the perspective of Family Systems Theory (Bowen, 1978; Cox and Paley, 1997; Minuchin, 1985), the family is conceptualized as an interconnected system in which the functioning of one member or subsystem is related to the functioning of the entire unit. Within this framework, grandparent involvement represents not merely a caregiving arrangement but also a systemic input that operates within the broader family context. The extent to which grandparent involvement intensity is associated with children’s social competence and subsequent behavioral outcomes may depend critically on the overall functional quality of the family system, including family cohesion, communication patterns, emotional bonding, and the family’s capacity to adapt to caregiving demands (Luo et al., 2020). When the family system functions adaptively, grandparent involvement may be associated with greater family strength, lower parental stress, and a supportive emotional climate that is related to children’s acquisition of social skills. Conversely, when the overall family system exhibits low cohesion or poor communication, the addition of intensive grandparent involvement may be associated with coordination challenges across subsystems and with lower consistency and richness of social learning opportunities available to the child (Jo-Pei et al., 2025). Theoretically, high-intensity grandparent involvement may redistribute children’s daily time away from exclusive parent-child interactions, which are uniquely rich in contingent feedback, emotion coaching, and social referencing, processes that are critically important for the development of social competence. Consistent with this theoretical logic, children’s social competence is hypothesized to constitute an important explanatory pathway linking family caregiving environments to child adjustment. Notably, the present study tests the indirect association via social competence but does not directly measure the hypothesized reduction in parent-child interaction time.

Accordingly, this study incorporates social competence as a mediator and family functioning as a moderator to propose a moderated mediation model (Figure 1). We aim to answer: (1) whether grandparent involvement is indirectly associated with children’s emotional and behavioral problems through social competence; and (2) whether family functioning moderates the relationship between grandparent involvement and social competence.

**FIGURE 1:**
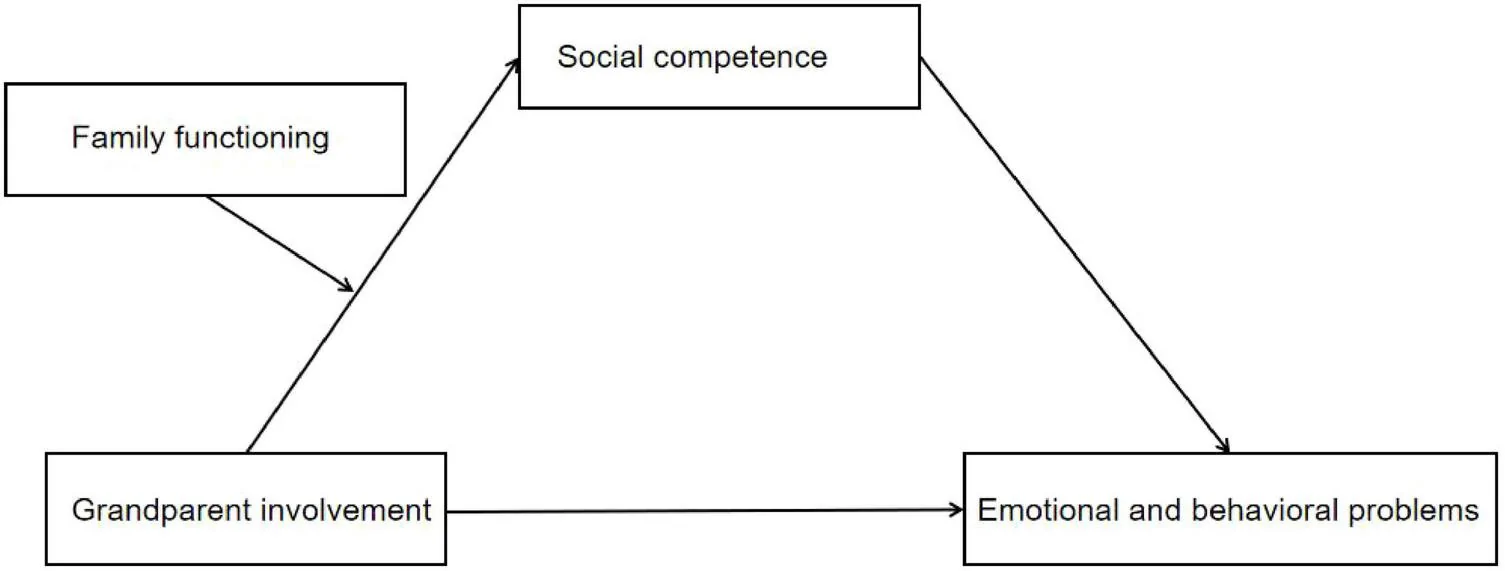
Conceptual model of this study.

## 2 Theoretical basis and research hypotheses

### 2.1 Theoretical basis

Family Systems Theory and the Bioecological Model serve as the overarching theoretical frameworks for this investigation. From a systemic perspective, the family operates as an interdependent unit where macro structural configurations correlate with the overall developmental context. When grandparents assume a substantial quantity of caregiving responsibilities, the daily household structure shifts from a traditional dyadic model to a multigenerational network. The Bioecological Model posits that early development is fundamentally linked to proximal processes, which are defined as the regular and extended daily interactions between children and their immediate caregivers (Bronfenbrenner and Morris, 2007). Accordingly, the substantial amount of time and frequent contact inherent in high intensity grandparent involvement are systematically associated with children’s developmental outcomes. Based on this, the present study examines how grandparent involvement is associated with early childhood developmental indicators.

### 2.2 The association between grandparent involvement and emotional and behavioral problems

In grandparent involvement scenarios, the deep intervention of grandparents is associated with a close intersection between the relationship system of the nuclear family and the broader intergenerational system, which is accompanied by a dynamic adjustment of family roles and rules (Bailey et al., 2009). Theoretically, when grandparent involvement accounts for a substantial proportion of the daily routine, children may spend correspondingly more time in domestic environments supervised primarily by older adults. This structural reallocation of time would logically correspond to a reduction in exclusive one-on-one interactions between parents and children. Extant literature demonstrates that the quantity of parental time investment is a significantly stronger correlate of early social and behavioral development compared to the duration of grandparental supervision (Milovanska-Farrington, 2021; Wang et al., 2024). Parent and child interactions are uniquely associated with higher levels of mutual emotional attunement and complex social scaffolding (Easterbrooks et al., 2012; Sofri et al., 2025). Parental emotional availability, defined as the capacity of the dyad to share an emotionally healthy relationship, is identified as a foundational factor positively correlated with children’s acquisition of core regulatory capacities (Saunders et al., 2015). Consequently, even when grandparents devote substantial hours to daily domestic supervision, this extensive temporal input does not symmetrically replace the specific developmental correlates of primary parental care. This asymmetrical pattern of caregiving time is consistent with the positive association between a high volume of grandparent involvement and children’s emotional and behavioral difficulties, and with the view that the unique developmental benefits of primary parental involvement are structurally diluted (Fergusson et al., 2008; Li et al., 2022; Zhao et al., 2025), as the unique developmental benefits of primary parental involvement are structurally diluted. Based on this structural rationale, the current study proposes the following hypothesis:

**Hypothesis 1**: Grandparent involvement is significantly and positively associated with children’s emotional and behavioral problems.

### 2.3 The mediating role of social competence

The developmental capacity for children’s social competence does not emerge spontaneously; rather, its development is associated with the continuous accumulation of diverse social experiences within the family environment (Halberstadt et al., 2001; Morris et al., 2007). The statistical association between grandparent involvement intensity and social competence does not stem from any inherent deficits in grandparent involvement. Instead, it relates to a structural reallocation of children’s daily time across different caregiving contexts (Chen et al., 2011). As theorized by prior work, when grandparents assume a majority of daily care responsibilities, children’s time allocation patterns may undergo a systematic shift, which could logically correspond to a reduction in the duration and frequency of exclusive parent–child interactions (Goh and Kuczynski, 2010). Importantly, this reduction in interaction time does not equate to poorer guidance quality from grandparents. Instead, parents and grandparents occupy distinct positions within the family system, and parents typically serve as the primary attachment figures. Their long term and high frequency daily interactions are associated with a highly nuanced understanding of the child’s unique social strengths and vulnerabilities (Opie et al., 2021; Zeanah et al., 2011). Based on this profound emotional bond, parents are uniquely positioned to provide targeted social scaffolding tailored to the child’s specific circumstances. For instance, parents can offer attributional coaching when a child experiences peer rejection or draw upon a shared history of past experiences to guide the child through complex social dilemmas (Lunkenheimer et al., 2007). Although grandparents are fully capable of providing warm and appropriate guidance, the intergenerational relationship does not structurally encompass the same depth of accumulated interaction and daily shared experiences. This conclusion is not a value judgment regarding care quality; rather, it reflects an objective reality associated with the natural status of parents as primary attachment figures (Groh et al., 2014). Therefore, when extensive grandparent involvement structurally accounts for a larger proportion of daily time, the total volume of highly personalized and attachment based social guidance that the child receives is objectively lower. Based on this structural rationale, the current study proposes the following hypothesis:

**Hypothesis 2**: Social competence mediates the relationship between grandparent involvement and emotional and behavioral problems.

### 2.4 The moderating role of family functioning

According to Family Systems Theory, a household operates as an interdependent unit where the macro level characteristics of the system contextualize the statistical associations between specific subsystem inputs and individual outcomes (Minuchin, 1985; Olson, 2000). Therefore, while the quantitative duration of grandparent involvement is structurally related to children’s social competence, the magnitude of this statistical association varies systematically across different overarching household climates (Fosco and Grych, 2013). Family functioning captures this macro level climate, reflecting the global capacity of the household to maintain systemic cohesion, establish supportive communication networks, and coordinate daily routines as a unified whole (Walsh, 2003).

From this systemic perspective, when a substantial volume of nonparental care time is integrated into the daily routine, global family functioning serves as an essential contextual moderator that conditions this temporal arrangement (McHale and Fivaz-Depeursinge, 1999). In households characterized by high family functioning, the family system exhibits strong internal cohesion and reciprocal emotional support among all adult members. Within such resilient environments, even when grandparent involvement accounts for a large proportion of the daily time allocation, the overall household context remains highly coordinated. This background helps optimize the developmental value of the limited remaining periods of exclusive parent and child interactions (Conger et al., 2010). Consequently, a highly functional family climate acts as a systemic buffer, attenuating the negative structural association between extensive grandparent involvement time and the child’s social competence. Conversely, in households with low family functioning, the systemic environment lacks cohesion and competence. When high quantities of grandparent involvement occur in this fragmented macro context, any theoretically expected structural reduction in primary parental time may be accompanied by poor overarching coordination (Stocker et al., 2007). Therefore, in strict alignment with Family Systems Theory, this study posits that the overall functional quality of the family system critically moderates the primary structural associations within the caregiving environment.

**Hypothesis 3**: Family functioning moderates the association between grandparent involvement and children’s social competence. Specifically, higher family functioning attenuates the negative correlation between the quantity of grandparent involvement time and children’s social competence.

## 3 Research methods

### 3.1 Sample and procedure

This study employed a multi-time-point longitudinal design with three assessment waves spaced 1 week apart. Participants were recruited through an online survey platform Wenjuanxing using a convenience sampling approach. The survey was distributed to parents of children aged 3–7 years across China.

To reduce the influence of common method bias and to ensure data reliability, each family was asked to designate the primary caregiver, defined as the parent who was most familiar with the child’s daily life and emotional and behavioral status, to complete all questionnaires at all three time points. This single consistent informant approach was applied as a screening criterion to avoid non-independence issues that arise when both parents provide simultaneous responses. Thus, the same informant (predominantly the mother; see demographics below) reported on grandparent involvement and family functioning at T1, child social adaptability at T2, and child emotional and behavioral problems at T3, respectively. This temporal separation of predictor, mediator, and outcome measurements further reduces the risk of common method bias (Podsakoff et al., 2003).

Inclusion criteria were: (a) families with children aged 3–7 years; (b) families in which the primary caregiver was willing to participate in all three survey waves. Exclusion criteria included single-parent families and families in which the child had a diagnosed developmental or psychiatric disorder.

A total of 858 families completed the T1 survey, of which 729 provided complete responses across all three waves, yielding a retention rate of 85% and an attrition rate of 15%. Missing data were minimal and handled via listwise deletion. In the final sample (*N* = 729), categorical demographic variables were coded as follows: respondent identity was coded as 1 = father (*N* = 163, 21.5%) and 2 = mother (*N* = 596, 78.5%, *M* = 1.79, SD = 0.41); child gender was coded as 1 = boy (*N* = 381, 50.2%) and 2 = girl (*N* = 378, 49.8%, *M* = 1.50, SD = 0.50); respondent education was coded on an ordinal scale from 1 = junior high school or below (*N* = 230, 30.3%) to 5 = postgraduate degree or above (*N* = 46, 6.1%, *M* = 2.22, SD = 1.14); and child age was treated as a continuous variable ranging from 3.00 to 7.00 years (*M* = 4.82, SD = 1.11). Informed consent was electronically obtained from all participating parents prior to survey administration, and the study protocol received ethics approval from the Institutional Review Board.

### 3.2 Research tools

All items in this study were measured using a five-point Likert scale, but the anchor labels differed across scales. Specifically, the scales for Grandparent involvement, Family functioning, and Emotional and behavioral problems used anchors from 1 (“very inconsistent”) to 5 (“very consistent”). The Social Competence scale used anchors from 1 (“never”) to 5 (“always”).

Grandparent involvement (T1): This study used six items adapted from the China Family Panel Studies (CFPS) 2012 to assess grandparent involvement (Xie and Hu, 2014). All items measured the quantity or frequency of caregiving provided by grandparents, rather than the quality or nature of intergenerational interactions. The full set of six items includes: (1) “My child is mainly cared for by my parents or parents-in-law”; (2) “My parents or parents-in-law take care of my child on a daily basis”; (3) “My child spends most of the daytime with my parents or parents-in-law”; (4) “My parents or parents-in-law are responsible for picking up my child from kindergarten/school”; (5) “My parents or parents-in-law prepare meals for my child”; and (6) “My parents or parents-in-law put my child to bed.” Responses were rated on a five-point Likert scale ranging from 1 (“very inconsistent”) to 5 (“very consistent”), with higher scores indicating a greater extent of grandparent involvement for the child. In this study, the Cronbach’s α of the Grandparent Involvement Scale was 0.73.

Family functioning (T1): The General Functioning subscale of the McMaster Family Assessment Device (FAD-GF; Epstein et al., 1983) was used to assess overall family functioning. The scale consists of 12 items, originally rated on a 4-point Likert scale with some items reverse-scored. To adapt the measure for three-generation Chinese households, we modified the item wording by replacing the pronoun “We” with “Our family members” and rewording all items in a positive direction, with higher scores indicating better functioning. A sample item is: “Our family members can express feelings to each other.” Translation and back-translation procedures were conducted strictly following Brislin’s (1980) guidelines to guarantee cross-cultural equivalence. To maintain consistent response formatting across the full survey battery, the response scale was expanded from the original 4-point scale to a 5-point Likert scale (1 = “very inconsistent” to 5 = “very consistent”). Research demonstrates that converting 4-point scales to 5-point Likert formats improves scale sensitivity and variance without distorting item psychometric properties or factor loadings (Lozano et al., 2008; Preston and Colman, 2000). Confirmatory factor analysis supported a one-factor structure with excellent fit: χ^2^/df = 1.374, CFI = 0.996, TLI = 0.995, RMSEA = 0.022, RMR = 0.020, indicating good structural validity. The Chinese version of the FAD has demonstrated good reliability and validity in Chinese samples (Shek, 2001, 2002). In this study, Cronbach’s α was 0.94. While a Cronbach’s α exceeding 0.90 may occasionally signal item redundancy, it is typical for cohesive, highly unidimensional global constructs like the FAD-GF when administered to homogenous primary caregiver samples (Streiner, 2003).

Children’s Social competence (T2): Children’s interpersonal adaptation and social skills were measured using the Social Competence subscale of the Social Competence and Behavior Evaluation scale (SCBE-30; LaFreniere and Dumas, 1996), translated and validated for Chinese preschool populations by Liu et al. (2012). The subscale contains 10 items assessing prosocial behaviors, emotional expression, and peer cooperation; a sample item is “When conflicts occur with other children, the child can negotiate with them to resolve the conflict.” Primary caregivers rated each item on a 5-point Likert scale ranging from 1 (“never”) to 5 (“always”). The 5-point response format aligns with previous Chinese preschool adaptations of the SCBE-30, which confirmed strong factor stability and criterion validity (Liu et al., 2012). Higher mean scores indicate greater child social competence. In the present study, Cronbach’s α was 0.94. This high reliability reflects the strong internal consistency and conceptual coherence among the 10 prosocial items when rated by primary caregivers observing daily routines (LaFreniere and Dumas, 1996; Streiner, 2003).

Emotional and behavioral problems (T3): The parent-report Strengths and Difficulties Questionnaire (SDQ; Goodman, 1997) was used to assess children’s emotional and behavioral problems, completed by the primary caregiver. The outcome score was operationalized as the SDQ total difficulties score, calculated by summing the 20 items from the four difficulties subscales–emotional symptoms, conduct problems, hyperactivity/inattention, and peer relationship problems; the five prosocial items were excluded because they assess positive social functioning rather than problems (Goodman, 1997). The Chinese parent version has demonstrated acceptable reliability and validity in Chinese child samples (Du et al., 2008). The original SDQ uses a 3-point scale (0 = not true, 2 = certainly true), with some positively worded items requiring reverse coding. To maintain consistent scoring direction and reduce response errors, we adapted the scale by (1) adopting a 5-point Likert scale (1 = strongly disagree, 5 = strongly agree) and (2) rewording all items in the direction of difficulties, so that higher scores consistently indicate more problems and no reverse coding is needed. Similar adaptation or reverse-coding procedures have been applied in prior research (Sangha, 2025). Higher total scores therefore indicate more emotional and behavioral problems. In the present study, Cronbach’s α for the 20-item total difficulties score was 0.97, indicating excellent internal consistency. Although α-values above 0.90 may raise concerns about item redundancy, such high internal consistency is not uncommon for scales with highly interrelated items measuring a unidimensional construct, particularly when using a 5-point response format (Streiner, 2003; Tavakol and Dennick, 2011). To further evaluate the structural validity of the scale, we conducted a confirmatory factor analysis (CFA) on the four-factor model of the difficulties items. The results demonstrated good model fit: CFI = 0.997, TLI = 0.997, RMSEA = 0.017, RMR = 0.020, χ^2^/df = 1.231, with all items loading significantly on their respective factors (*p* < 0.001), supporting the structural validity of the scale despite the high alpha. It should be noted that this adapted version of the SDQ has not been independently validated in previous research. The CFA results reported in Table 1 and the high internal consistency (α = 0.97) observed in the present sample provide preliminary evidence for the structural validity and reliability of the adapted version within this study. However, independent validation in future samples is warranted.

**TABLE 1:** Results of confirmatory factor analysis.

Model		χ^2^	*df*	CFI	TLI	RMSEA	SRMR
Model1	Four-factor model	1616.35	1076	0.975	0.973	0.026	0.049
Model 2	Three-factor model 1	2335.982	1079	0.941	0.938	0.04	0.069
Model 3	Three-factor model 2	5906.901	1079	0.773	0.762	0.078	0.118
Model 4	Three-factor model 3	5778.494	1079	0.779	0.769	0.077	0.12
Model 5	Two-factor model	6092.06	1081	0.764	0.754	0.08	0.121
Model 6	One-factor model	9680.054	1082	0.595	0.578	0.104	0.143

Control variables: Given demographic characteristics, we used respondent gender, respondent education level, child gender, and child age as control variables in the data analysis.

### 3.3 Data analysis methods

This study used SPSS 27.0 for descriptive statistics, correlation analysis, and reliability testing, and used Model 7 in the SPSS PROCESS 4.0 macro to test the moderated mediation effect.

## 4 Results

### 4.1 Confirmatory factor analysis (CFA)

Prior to hypothesis testing, we conducted a series of confirmatory factor analyses (CFA) to examine the discriminant validity of the focal constructs. We specified a baseline four-factor model and compared it against a set of nested alternative models: three different three-factor models (combining different pairs of constructs), a two-factor model, and a one-factor model. The analyses were performed using Mplus 8.3. As shown in Table 1, the baseline four-factor model demonstrated excellent fit to the data (χ^2^ = 1616.350, df = 1076, CFI = 0.975, TLI = 0.973, RMSEA = 0.026, SRMR = 0.049). All fit indices met or exceeded the recommended criteria for good model fit (CFI/TLI ≥ 0.95, RMSEA ≤ 0.06, SRMR ≤ 0.08). More importantly, the four-factor model consistently outperformed all alternative models across every fit index. For instance, when any two constructs were combined into a single factor (the three-factor models), CFI values dropped to 0.941, 0.773, and 0.779, with corresponding increases in RMSEA and SRMR. The two-factor and one-factor models yielded even poorer fit (CFI = 0.764 and 0.595, respectively). Additionally, all standardized factor loadings in the four-factor model were significant at the *p* < 0.01 level. Taken together, these results provide strong empirical support for the distinctiveness of the four constructs. Detailed fit statistics are presented in Table 1.

### 4.2 Common method bias test

This study used a multi-time-point survey approach to avoid common method bias. Before data analysis, we conducted Harman’s single-factor test. The results showed that the first common factor explained 32.325% of the variance, which is below the critical value of 40%, indicating that serious common method bias was not present in the data.

### 4.3 Descriptive statistics and correlation analysis

Table 2 presents the descriptive statistics for each variable and the correlations among them. The results showed that grandparent involvement was significantly negatively correlated with children’s social competence and significantly positively correlated with children’s emotional and behavioral problems. Children’s social competence was significantly negatively correlated with children’s emotional and behavioral problems. Family functioning was significantly negatively correlated with grandparent involvement, significantly positively correlated with children’s social competence, and significantly negatively correlated with children’s emotional and behavioral problems.

**TABLE 2:** Mean, standard deviation and correlation coefficient of variables.

Variables	M	SD	1	2	3	4	5	6
1. Grandparent involvement	2.34	0.64	–	–	–	–	–	–
2. Family functioning	3.24	0.83	−0.16**					
3. Social competence	3.25	0.93	−0.34**	0.35**				
4. Emotional and behavioral problems	2.73	0.92	0.31**	−0.33**	−0.47**			
5. Respondent education level	2.20	1.14	0.03	−0.01	0.04	−0.03		
6. Age of children	4.81	1.10	−0.05	−0.01	0.02	−0.07	0.07*	

*N* = 729. **p < 0.01; *p < 0.05.

### 4.4 Hypothesis testing

We used the PROCESS macro (Hayes, 2013) for SPSS to conduct mediation and moderated mediation analyses. For mediation analysis, we employed Model 4; for moderated mediation analysis, we employed Model 7. Parameter estimation used bootstrapping with 5,000 resamples; a 95% confidence interval that did not include zero indicated statistical significance. Prior to analysis, continuous variables were mean-centered to reduce multicollinearity in the interaction terms (Aiken, 1991). All regression coefficients reported are unstandardized coefficients (B) from the PROCESS output. Results are shown in Tables 3–5.

**TABLE 3:** The results of the regression analysis.

Variables	M (social competence)					Y (emotional and behavioral problems)				
	B (SE)	t	*p*	LLCI	ULCI	B (SE)	t	*p*	LLCI	ULCI
X (grandparent involvement)	−0.35 (0.05)	−7.22	<0.001	−0.45	−0.26	0.25 (0.05)	5.22	<0.001	0.16	0.35
W (family functioning)	0.29 (0.04)	7.52	<0.001	0.21	0.36	–	–	–	–	–
X × W	0.12 (0.06)	2.00	0.05	0.00	0.24	–	–	–	–	–
M (social competence)	–	–	–	–	–	−0.40 (0.03)	−11.51	<0.001	−0.46	−0.33
F	45.77					107.45				
R^2^	0.16					0.23				

*N* = 729. Unstandardized coefficients (B) are reported with standard errors in parentheses. LLCI, lower limit of 95% confidence interval; ULCI, upper limit of 95% confidence interval.

**TABLE 4:** The mediating role of social competence between grandparent involvement and children’s emotional and behavioral problems.

Effect type	Pathway	Effect	Boot SE	Boot LLCI	Boot ULCI
Total effect	GI → EBP	0.42	–	–	–
Direct effect	GI → EBP (controlling for SC)	0.25	0.05	0.16	0.35
Indirect effect	GI → SC → EBP	0.17	0.02	0.13	0.21

*N* = 729. GI, grandparent involvement; SC, social competence; EBP, emotional and behavioral problems. Bootstrap resampling = 5,000.

**TABLE 5:** Moderated mediation effect: the moderating role of family functioning.

Effect type	Statistic	B	Boot SE	Boot LLCI	Boot ULCI
Interaction effect	X × W → SC	0.12	0.06 (SE)	0.00	0.24
Index of moderated mediation	X × W × M	−0.05	0.02	−0.09	−0.01
Conditional indirect effects	Low family functioning (*W* = M − 1SD)	0.18	0.03	0.13	0.23
	Mean family functioning (W = M)	0.14	0.02	0.10	0.18
	High family functioning (*W* = M + 1SD)	0.10	0.03	0.04	0.16

*N* = 729. X, grandparent involvement; W, family functioning; SC, social competence; M, mediator (social competence); Y, emotional and behavioral problems. Bootstrap resampling = 5,000.

Hypothesis 1 proposed that grandparent involvement is positively associated with children’s emotional and behavioral problems. As shown in Table 3, grandparent involvement was significantly positively associated with children’s emotional and behavioral problems (*B* = 0.25, SE = 0.05, *t* = 5.22, *p* < 0.001), supporting Hypothesis 1.

Hypothesis 2 predicted that social competence mediates the relationship between grandparent involvement and children’s emotional and behavioral problems. As shown in Table 3, grandparent involvement was significantly negatively associated with children’s social competence (*B* = −0.35, SE = 0.05, *t* = −7.22, *p* < 0.001), and children’s social competence was significantly negatively associated with children’s emotional and behavioral problems (*B* = −0.40, SE = 0.03, *t* = −11.51, *p* < 0.001). As shown in Table 4, the indirect effect of grandparent involvement on children’s emotional and behavioral problems through social competence was significant (*B* = 0.15, Boot SE = 0.02, 95% CI [0.13, 0.21]). The total effect was *B* = 0.42 (SE = 0.05, *p* < 0.001), and the direct effect remained significant (*B* = 0.25, Boot SE = 0.05, 95% CI [0.16, 0.35]). The indirect effect accounted for approximately 39.74% of the total effect. These results support Hypothesis 2, indicating that social competence partially mediates the relationship between grandparent involvement and children’s emotional and behavioral problems.

Hypothesis 3 predicted that family functioning moderates the relationship between grandparent involvement and children’s social competence. As shown in Table 3, the interaction term between grandparent involvement and family functioning was significantly associated with children’s social competence (*B* = 0.12, SE = 0.06, *t* = 2.00, *p* = 0.05). Simple slope analysis showed that when family functioning was low (one SD below the mean), grandparent involvement was negatively associated with social competence with a relatively stronger magnitude (*B* = −0.46, SE = 0.07, *t* = −6.67, *p* < 0.001); when family functioning was high (one SD above the mean), the negative association between grandparent involvement and social competence was weaker (*B* = −0.25, SE = 0.07, *t* = −3.36, *p* = 0.001) (see Figure 2). This pattern indicates that higher family functioning attenuates the negative association between grandparent involvement and children’s social competence, supporting Hypothesis 3.

**FIGURE 2:**
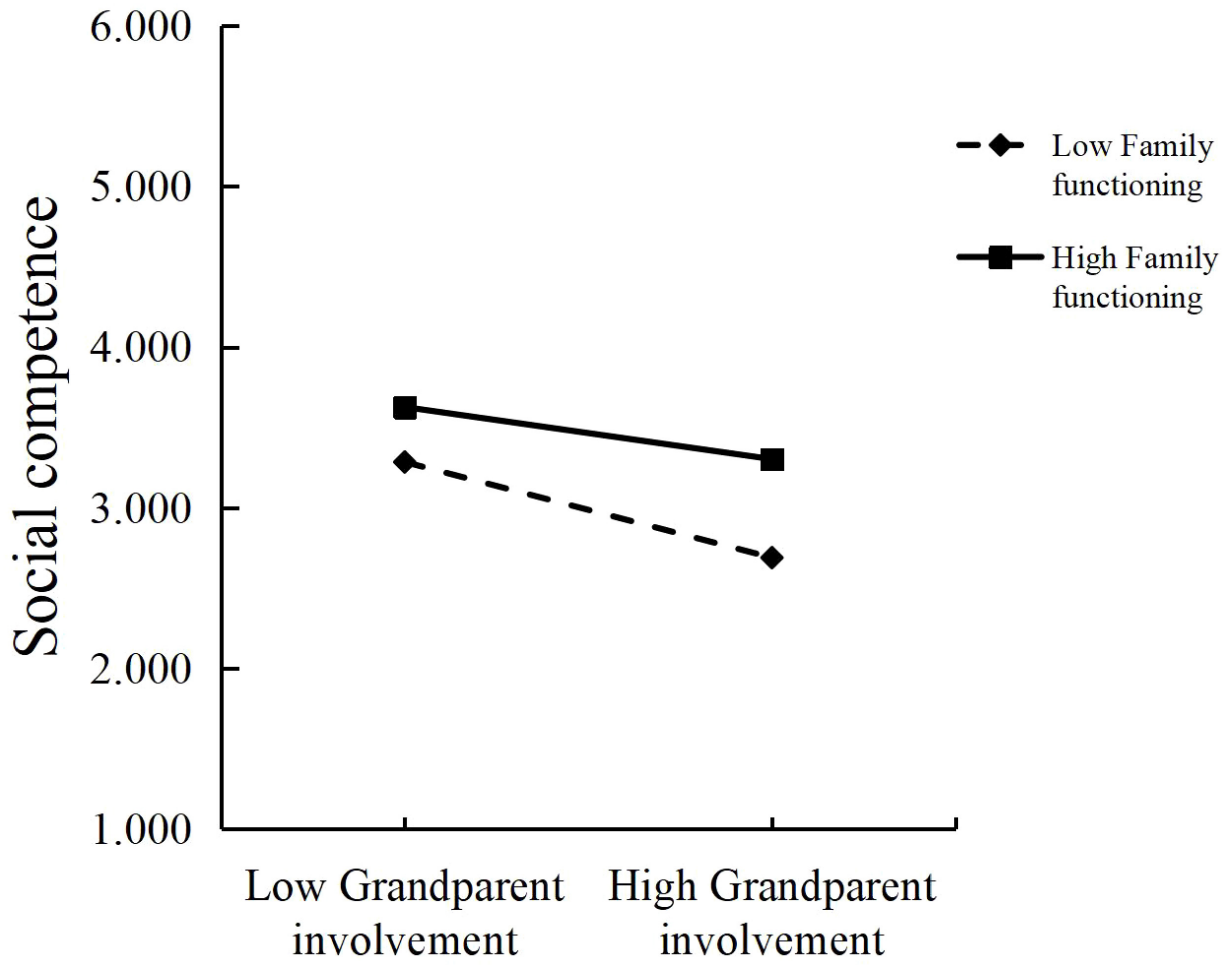
The moderating association of family functioning on the relationship between grandparent involvement and children’s social competence.

To further examine the moderated mediation effect, we computed the index of moderated mediation. As shown in Table 5, the index was −0.05 (Boot SE = 0.02, 95% CI [−0.09, −0.01]), which did not contain zero, indicating a significant moderated mediation effect. The conditional indirect effects showed a stable decreasing pattern across levels of family functioning: at low (*W* = −0.84), mean (*W* = 0), and high (*W* = 0.84) levels of family functioning, the conditional indirect effects were 0.18 (95% CI [0.13, 0.23]), 0.14 (95% CI [0.10, 0.18]), and 0.10 (95% CI [0.04, 0.16]), respectively. These results indicate that the indirect effect of grandparent involvement on children’s emotional and behavioral problems through social competence becomes weaker as family functioning increases, supporting the moderated mediation mechanism.

## 5 Discussion and implications

### 5.1 Discussion

First, this study observed a significant positive prospective association between the quantity of grandparent involvement and children’s emotional and behavioral problems. This finding aligns with prior empirical literature (Bramlett and Blumberg, 2007; Xu et al., 2022) indicating that higher levels of grandparent involvement are prospectively linked to elevated risks of child emotional distress and behavioral dysregulation. Grounded in Family Systems Theory and the Bioecological Model, this pattern is theoretically consistent with a structural “crowding-out” mechanism and reduced parental availability. That is, one plausible interpretation is that when extensive grandparental supervision time substitutes for daily primary parental involvement, such structural substitution could dilute children’s ongoing opportunities to internalize core emotional regulation capacities. Nevertheless, it is important to acknowledge that the present study did not directly measure parent–child interaction time or parental emotional availability. Thus, while our findings are consistent with the crowding-out account, this mechanism remains theoretically inferred rather than empirically tested in our data. Future research should directly measure time use and interaction quality to evaluate this explanatory pathway.

Second, the findings demonstrate that children’s social competence serves as a temporally ordered statistical mediator in the prospective relationship between grandparent involvement and children’s emotional and behavioral problems. Previous research has primarily examined direct associations with cognitive outcomes or examined behavioral links in relation to qualitative parenting styles and intergenerational friction (Jampaklay, 2006). In contrast, our findings emphasize that when grandparents assume a substantial share of daily care, the structural reduction in exclusive parent-child interaction time is associated with reduced continuous access to targeted social scaffolding uniquely provided by primary attachment figures, including attributional coaching, emotion processing, and conflict resolution guidance. When children exhibit lower social competence, they face greater adaptive challenges during interpersonal stress, which is prospectively associated with higher internalizing (e.g., anxiety, withdrawal) and externalizing (e.g., aggression, oppositional behavior) difficulties (Compas et al., 2001). This identifies social competence as a key psychological resource through which structural caregiving time allocation is prospectively associated with children’s adjustment.

Third, family functioning was found to significantly moderate the prospective link between grandparent involvement and children’s social competence. Specifically, in highly functional families, the negative prospective association between the quantity of grandparent involvement and children’s social competence was weaker; conversely, in low-functioning families, this inverse association was stronger. From a family systems perspective, this conditional pattern indicates that the overarching functional climate of the household is related to whether the prospective developmental risks associated with parental time crowding-out are stronger or weaker. In highly cohesive and communicative family environments, parents and grandparents coordinate routines effectively, and this coordination may help parents make greater use of the developmental quality of the interaction periods they do have with their children, which in turn may be associated with children internalizing adaptive social norms. In contrast, within low-functioning families characterized by fragmented communication, high grandparent involvement is accompanied by poor overall coordination and fewer consistent opportunities for children to consolidate social skills (Bai et al., 2023). Although the interaction effect size was modest, this conditional pattern carries crucial theoretical and practical value, suggesting that whole-family functional quality serves as a vital contextual moderator of the prospective developmental correlates of grandparent involvement.

### 5.2 Theoretical implications

This study has three main theoretical implications.

First, this study systematically applied family systems theory to the context of grandparent involvement and empirically tested the theoretical pathways. Previous studies have mostly discussed the consequences of grandparent involvement or the main effects of family functioning in isolation, rarely integrating both into a single theoretical model. By testing the moderating role of family functioning, this study revealed how the overall functional state of the family is related to the direction and strength of the association between grandparent involvement and children’s social competence, which expands the theoretical boundaries of family systems theory in the context of multigenerational childcare arrangements and high-intensity grandparent involvement.

Second, this study introduced social competence as an indirect pathway variable, which contributes to a more process-based understanding of how grandparent involvement is associated with children’s emotional and behavioral problems. Unlike previous studies that focused mainly on direct predictions of cognition or behavioral habits, this study identified an indirect association pattern involving grandparent involvement, social competence, and emotional and behavioral problems. This finding connects parenting styles within the family to children’s adaptive performance in the external social environment, enriching the theoretical connotation of the associations of grandparent involvement and responding to calls in the literature to examine mediating mechanisms.

Third, by testing the moderating role of family functioning, this study broke through the simplistic interpretation of the associations of grandparent involvement. The results show that grandparent involvement is not always negative; its association varies according to the overall functional level of the family system. This finding provides empirical support for the core proposition of context dependence in family systems theory and offers a theoretical framework for future research to shift from asking “whether there is an association” to “when and how the association occurs.”

### 5.3 Practical implications

Based on the findings of this study, we offer the following practical considerations. Our evidence is observational in nature, establishing temporally ordered associations rather than causal association. Therefore, the suggestions below should be interpreted as potentially promising intervention targets that warrant rigorous testing in future experimental or quasi-experimental research, rather than as established guidelines for immediate implementation.

First, our findings suggest that family functioning may represent a plausible entry point for support services in grandparent involved families. Family functioning moderated the association between grandparent involvement and children’s social competence. This pattern is consistent with the possibility that strengthening family interaction patterns could be related to weaker negative correlates of grandparent involvement. On this basis, we hypothesize that community based or school based services targeting family functioning, such as intergenerational communication workshops or role clarification sessions, might be beneficial, particularly for families with lower functioning. However, the effectiveness of such interventions remains to be empirically tested. We encourage future studies to evaluate whether improving family functioning is associated with better child outcomes.

Second, social competence emerged as a significant indirect pathway in the association between grandparent involvement and emotional and behavioral problems. Enhancing children’s social skills may therefore be another promising intervention target. For instance, group based social skills training, including role playing peer interaction, conflict resolution, and emotional expression exercises, might help children in grandparent involved families develop greater interpersonal competence. Additionally, encouraging parents and grandparents to reduce overprotection and create opportunities for children to make independent decisions and engage in peer activities could potentially support social skill development. Nevertheless, these strategies are speculative at this stage. Their efficacy should be examined in future intervention studies that directly test whether improvements in social competence are associated with subsequent reductions in emotional and behavioral difficulties.

In sum, we propose that family functioning and children’s social competence represent theoretically grounded and empirically associated targets that merit further investigation as potential components of preventive or early intervention programs. We emphasize that these proposals are offered as hypotheses for future intervention research, not as confirmed practice recommendations. We hope they will stimulate rigorous experimental evaluations to determine whether modifying these factors produces anticipated benefits for children in grandparent involved families.

### 5.4 Limitations and future research

Although this study offers valuable theoretical contributions and practical implications, several limitations should be acknowledged to guide future research.

First, although data were collected across three time points to establish temporal ordering, the 1-week interval between waves was short, and mediator and outcome variables were not measured repeatedly across waves. Without controlling for baseline levels of social competence and emotional/behavioral difficulties at T1, temporal sequence alone cannot confirm causal mediation or rule out reverse causality–such as child behavioral difficulties evoking greater grandparent involvement or contributing to family dysfunction. While the study is not purely cross-sectional, its temporal structure remains insufficient to support strong causal inferences. Additionally, the observed interaction effect between grandparent involvement and family functioning, though statistically significant, was modest in magnitude, and the family functioning measure may have diluted the moderating effect; future research using more targeted intergenerational care measures could yield clearer estimates. Future research should employ longitudinal panel designs over longer time spans with baseline controls to rigorously evaluate causal pathways.

Second, all focal constructs were assessed via parent reports, which is associated with potential shared-informant bias. A parent who perceives family functioning unfavorably may also be more likely to view the child as socially or behaviorally problematic. The three-wave design with short intervals does not fully eliminate these common-method concerns. Incorporating teacher ratings or direct observational measures in future studies would be associated with substantially stronger construct validity.

Third, the sample was drawn exclusively from Chinese families and measured grandparent involvement primarily in terms of quantitative caregiving frequency without distinguishing specific qualitative arrangements (e.g., custodial grandparenting vs. partial grandparental care, full-time vs. part-time). Future cross-cultural studies should incorporate more nuanced operationalizations of grandparenting to test the generalizability and contextual variations of these findings. Additionally, while we invoked a crowding-out mechanism as the theoretical rationale, we did not directly measure parent-child interaction time or parental emotional availability; thus, this mechanism remains theoretically inferred rather than empirically tested in the present study. Future research should include direct assessments of time use and interaction quality to evaluate this proposed pathway.

## Data Availability

The raw data supporting the conclusions of this article will be made available by the authors, without undue reservation.
